# Prospects of mHealth Services in Bangladesh: Recent Evidence from Chakaria

**DOI:** 10.1371/journal.pone.0111413

**Published:** 2014-11-06

**Authors:** Fatema Khatun, SMA. Hanifi, Mohammad Iqbal, Sabrina Rasheed, M. Shafiqur Rahman, Tanvir Ahmed, Shahidul Hoque, Tamanna Sharmin, Nazib Uz Zaman Khan, Shehrin Shaila Mahmood, David H. Peters, Abbas Bhuiya

**Affiliations:** 1 International Centre for Diarrhoeal Disease Research, Bangladesh (icddr,b), 68 Shaheed Tajuddin Ahmed Sarani, Mohakhali, Dhaka-1212, Bangladesh; 2 School of Public Health and Community Medicine, The University of New South Wales, Kensington, New South Wales 2052, Australia; 3 Asia-Pacific ubiquitous Healthcare Research Centre, School of Information Systems, Technology and Management, Australian School of Business, The University of New South Wales, Kensington, New South Wales 2052, Australia; 4 Institute of Statistical Research and Training, University of Dhaka, Dhaka-1000, Bangladesh; 5 Department of International Health, The Johns Hopkins Bloomberg School of Public Health, 615 N. Wolfe Street, Baltimore, Maryland 21205, United States of America; The National Institute for Health Innovation, New Zealand

## Abstract

**Introduction:**

Bangladesh has a serious shortage of qualified health workforce. The limited numbers of trained service providers are based in urban areas, which limits access to quality healthcare for the rural population. mHealth provides a new opportunity to ensure access to quality services to the population. A recent review suggested that there are 19 mHealth initiatives in the country. This paper reports findings on people's knowledge, perception, use, cost and compliance with advice received from mHealth services from a study carried out during 2012–13 in Chakaria, a rural sub-district in Bangladesh.

**Methods:**

A total of 4,915 randomly-chosen respondents aged 18 years and above were interviewed.

**Results:**

Household ownership of mobile phones in the study area has increased from 2% in 2004 to 81% in 2012; 45% of the respondents reported that they had mobile phones. Thirty-one percent of the respondents were aware of the use of mobile phones for healthcare. Very few people were aware of the available mHealth services. Males, younger age group, better educated, and those from richer households were more knowledgeable about the existing mHealth services. Among the respondents who sought healthcare in the preceding two weeks of the survey, only 2% used mobile phones for healthcare. Adherence to the advice from the healthcare providers in terms of purchasing and taking the drugs was somewhat similar between the patients who used mobile phone for consultation versus making a physical visit.

**Conclusions:**

The high penetration of mobile phones into the society provides a unique opportunity to use the mHealth technology for consulting healthcare providers. Although knowledge of the existence of mHealth services was low, it was encouraging that the compliance with the prescriptions was almost similar for advice received through mobile phone and physical visits. The study revealed clear indications that society is looking forward to embracing the mHealth technology.

## Introduction

Bangladesh continues to be one of the 57 countries with a serious shortage of trained doctors, paramedics, nurses and midwives despite attempt for increased production in the recent years [1]. Bangladesh Health Watch reported in 2008 that there were only three physicians and two nurses per 10,000 population [2]. The presence of the limited number of qualified service providers is highly urban-centred, with 86% of the physicians; and 75% of the nurses and dentists are based in urban areas where only 20% of the population lives [2]. Rural health service delivery is dominated by informally-trained village doctors and drug-sellers practicing modern medicine. Contact rate with qualified healthcare providers is low and highly inequitable in terms of socioeconomic status of the patients and place of residence [3]. The first port of call for the poor is the informally trained village doctors and drug retailers who make their living by selling drugs. They quite often use more than necessary and inappropriate drugs for treating patients [3], [4]. Thus for the shortage of qualified healthcare providers and their geographic mal-distribution, access to quality healthcare by the people in general and the disadvantaged section of people in particular is constrained. Given the current level of production of trained health workforce, it is very unlikely that the situation will change drastically in the near future.

Under these circumstances, the use of Information and Communication Technology (ICT), e.g.mobile phone connectivity provides a new opportunity to increase access to quality healthcare for the population [5], [6]. A recent review of the growth of mHealth services in Bangladesh documented 19 initiatives involving mHealth services from call centres sending messages from health authorities to subscribers. Details of these initiatives can be seen elsewhere [7]. Making optimum use of the ICT will depend on the development of appropriate programmes informed by research on peoples' perception about the use of ICT and mobile phone, and making use of the feedback from the user on the existing services. A continuous monitoring of the change in perception about quality and adequacy of the service, along with problems faced by the people in accessing and using the services will help further improve the existing services. Despite the potential of this technology in providing healthcare services, a systematic assessment of the people's knowledge of, attitude about, and use of mobile phone and mHealth services has been lacking in the literature. Keeping this in mind this paper presents findings from a survey on mobile phone and mHealth which was carried out in a rural area of Bangladesh. It is expected that the findings will be useful in improving the existing ones and designing new programmes.

## Methods and Materials

### Study Area

The study was carried out in eight unions of Chakaria upazilla (sub-district), under Cox's Bazar district in the south-east costal area of Bangladesh where icddr,b has been running a Health and Demographic Surveillance System (HDSS) since 1999 [8]. The surveillance covers 118,335 residents living in 20,124 households. The population density is 782 individuals/km^2^, considerably lower than national average of 939/km^2^. The population comprises mainly Muslims (90%), a small number of Hindus (7%), and Buddhists (3%). The Bangalees constitute 99% of the households, and others are from an ethnic minority group called *Mogh* (*Rakhain*). The main economic activities in the area have been agriculture, forestry and sea-fishing. Typographically Chakaria is similar to other coastal areas in Chittagong district but different than rest of the country. Chakaria is a relatively low-performing area in terms of health and development indicators compared to areas of the central and western part of the country [9].

The availability of health services in the area is typical of other rural areas with public and private healthcare services with a very strong presence of informal healthcare providers [10]. icddr,b has been working with a selected number of communities since 1994 mainly in the area of primary healthcare with direct involvement in running six village health posts established and managed by the community. The icddr,b healthcare providers also have been available for consultations through mobile phones for the last 10 years. The government services included an upazilla health complex as the highest level of primary care, with seven family welfare centres, one rural dispensary, 14 community clinics at the lowest level [8]. There are three private clinics with the facility of general surgery [10]. During the study period, the villagers had access to mHealth services offered by a private company at the national level, which allowed consultation from physicians in the call centres. In addition, the government physicians of the upazilla health complex were also available for consultation through mobile phones. There was also a special service from Telemedicine Referral Centre Limlited, a private call centre, for a selected group of village doctors who participated in an icddr,b programme for the improvement of quality of their services [11].

### Study design

A total of 62,459 household members aged 18 years and above living in 20,124 households in Chakaria HDSS area in November 2012 formed the sampling frame for the survey. Because of unequal distribution of the population by both age and sex [8], a stratified sampling scheme with proportional allocation was employed to determine the sample-size for the survey. Stratification was done by separating males and females based on the age groups: 18–29, 30–39, 40–49, and 50 completed years and above. In the absence of any data on the level of knowledge about mobile phone based consultation services, a value of 50% was used for calculating sample-size separately for males and females for each of the age-groups, to have a reliable estimate of proportion of respondents having knowledge, using the services, and having a positive attitude towards mHealth with 95% confidence. This gives 384 (approximately 400) samples for each group. The sample-size from each of the age groups was calculated by using the sampling fraction (400/population of the age-group with lowest population size) multiplied by number of population in the age-group. This has resulted in a sample of 400 or more number of respondents in each age-group, with a total of 5152 respondents. Finally one respondent from each household was randomly chosen from the HDSS database. This sampling design has made the total sample self-weighted, making data analysis straight forward.

Data were collected by using a questionnaire developed in Bangla language which was duly pretested before finalization. Most of the questions had the provisions of recording pre-coded and open answers. Fifteen experienced female interviewers were recruited for the survey and were given a three day training on the survey questionnaire. Two experienced supervisors looked after the data-collection process. For quality control of the data collection, the supervisors re-visited 5% of the households chosen randomly within 2 days of data collection by the field workers to recollect data on some of the key questions. The supervisors and the relevant field workers together sorted out any inconsistencies in the collected data immediately making additional field visits if required. The survey was conducted during November 2012 to April 2013.

### Ethical consideration

Ethical Review Committee of the International Centre for Diarrhoeal Disease Research, Bangladesh provided approval for the project. Informed written consent was taken from all interviewees, and confidentiality and anonymity were ensured.

### Variables

The questionnaire included background characteristics of the respondents, ownership of mobile -phones, knowledge about the use of mobile phone for health care, various mHealth services, care seeking, cost of health care, preference between physical visit and telephone consultation, attitude toward mHealth, satisfaction with health care consultation through mobile phone, and socio-economic and demographic characteristics of individuals and households.

### Data analysis

Different answers to open-ended questions for all variables were listed, and every answer was assigned a numerical code for computer entry and processing. Exploratory analysis of data including summary statistics, graphs, and cross tabulation was carried out. For each proportion, 95% confidence interval (CI) was calculated based on exact binomial distribution. In some cases, test for statistical significance (parametric or non-parametric when required) was performed.

## Results

The survey team could successfully interview 1,964 male and 2,951 female respondents through repeated visits. Males were hard to reach for interviews for their absence in the household during daytime;- three repeated visits were made to minimize the rate of non-response. Finally, 89.8% males and 99.6% females could be interviewed.

### Characteristics of the respondents

The background characteristics of the respondents are presented in Table 1. The distribution of the background characteristics of the respondents is somewhat similar to that of the population aged 18 years and above in the study area [8] with an exception for distribution of sex.

**Table 1:** Characteristics of respondents.

Characteristics	% of the total respondents N = 4,915	95%CI
*Age (years)*		
<30	36.4	(34.8–37.5)
30–39	27.3	(25.9–28.4)
40–49	16.2	(15.0–17.1)
50+	20.1	(18.8–21.1)
*Sex*		
Male	39.9	(38.6–41.3)
Female	60.0	(58.7–61.4)
*Occupation*		
Household	46.8	(45.4–48.2)
Formal employment and business	9.2	(8.4–10.1)
Farming	6.2	(5.5–6.9)
Menial labour	22.1	(20.9–23.3)
Unemployed	15.7	(14.7–16.7)
*Education (years of schooling)*		
None	48.1	(46.7–49.5)
1–5	29.3	(28.0–30.6)
6+	22.6	(21.4–23.8)

### Ownership of mobile phone

**Figure 1:**
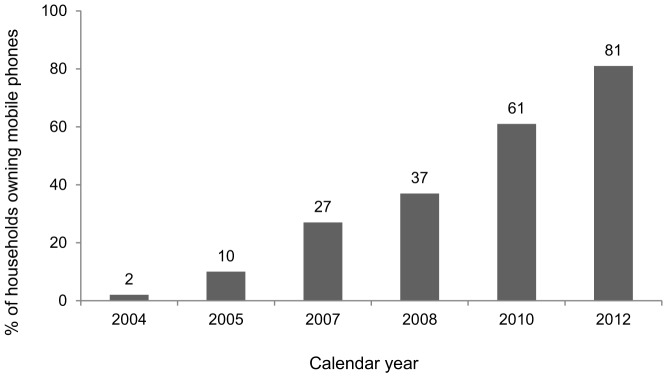
Household mobile phone ownership.

In the survey area, 45.5% of the respondents reported that they had a mobile phone (Table 2). 81% of the households had at least one phone. Data from the HDSS, independent of the present survey, recorded a steady increase in household ownership of mobile phones from 2% in 2004 to 81% in 2012 (Figure 1). The ownership of mobile phone was higher among males, younger age-groups, people from richer households, and the more-educated persons compared to females, those who were over 50 years of age, people from poorer households, and people with no education (Table 2).

**Table 2:** Ownership of mobile phone by background characteristics.

Characteristics	Number of respondents	% of respondents having a mobile phone	95% CI	Statistical significance
*Sex*				p = 0.000
Male	1964	61.76	(59.6–63.9)	
Female	2951	34.40	(32.7–36.1)	
*Age (years)*				p = 0.000
<30	1791	49.92	(47.6–52.3)	
30–39	1344	56.10	(53.4–58.8)	
40–49	794	43.70	(40.2–47.2)	
50+	986	23.63	(21.0–26.4)	
*Asset quintile*				p = 0.000
Poorest	1005	19.20	(16.8–21.7)	
2	973	35.97	(32.9–39.1)	
3	1002	48.10	(44.9–51.2)	
4	954	53.88	(50.7–57.1)	
Richest	981	70.23	(67.3–73.1)	
*Education (years of schooling)*				p = 0.000
None	2335	31.31	(29.4–33.2)	
1–5	1462	51.98	(49.4–54.6)	
6+	1112	66.10	(63.2–68.8)	
Total	4915	45.33	(43.9–46.7)	

### Knowledge of use of mobile phone for healthcare

In the study area, 31% (1,537) of the total respondents reported that they knew that mobile phone is being used for healthcare. Among the respondents who knew about the use of mobile phone for healthcare, knowledge about the use of mobile phone for healthcare was higher among males (39%) than females (26%) (Table 3). Only 1% knew about health helpline/call centres. Respondents perceived that the more educated and knowledgeable people tended to use mobile phone for healthcare compared to the less educated and less knowledgeable people.

**Table 3:** Awareness about the use of mobile phone for health care services.

Nature of awareness	% based on the respondents who knew about the service (N = 1,537)	95% CI
*Possible uses of mobile phone in health care:*		
Direct consultation	79.9	(77.8–81.9)
Appointment	11.8	(10.2–13.5)
Knowing availability	4.6	(3.6–5.8)
Clarification about prescription	3.2	(2.4–4.2)
Requesting home visit	0.5	(0.2–0.9)
*How people use mobile phone for health care?*		
To call MBBS doctor	69.5	(67.1–71.8)
To call village doctor	27.4	(25.2–29.7)
To call helpline	0.9	(0.4–1.4)
To call hospital/clinic	0.7	(0.3–1.2)
SACMO/MA/CHCP	0.1	(0.01–0.4)
Others	1.4	(0.8–2.1)
*Perception about who uses mHealth services:*		
Educated/knowledgeable	44.2	(41.7–46.7)
Upper class	11.1	(9.5–12.7)
Middle class	5.3	(4.2–6.5)
Lower class	4.5	(3.5–5.6)
Who has connection with health care providers	24.0	(21.9–26.2)
Employed/busy people	2.3	(1.5–3.2)
All types of people	7.9	(6.6–9.3)
Others	1.8	(1.2–2.5)

SACMO  =  Sub-Assistant Community Medical Officer.

MA  =  Medical Assistant.

CHCP  =  Community Health Care Provider.

### Knowledge of existing mHealth services

In terms of knowledge of the existing mHealth services very few people were aware of the services available through upazilla health complex, an initiative of the Ministry of Health or availability of special call centre numbers to call for health care (Table 4). Males, younger age- group, the better educated, and those from richer households were more knowledgeable about the existing mHealth services compared to females, those who were over 50 years of age, those who had no education and people from the poorest households (Table 4).

**Table 4:** Knowledge of existing mHealth services.

Variable	N	Percent of respondents who knew about the existing services			
		Special number % (95% CI)	Statistical significance	Upazila Health Complex % (95% CI)	Statistical significance
Existence of mHealth services	4,915	3.9 (3.3–4.4)		5.0 (4.4–5.7)	
*Sex*			p = 0.00		p = 0.000
Male	1,964	7.0 (5.9–8.2)		7.6 (6.5–8.9)	
Female	2,951	1.7 (1.3–2.3)		3.2 (2.6–3.9)	
*Age (years)*			p = 0.000		p = 0.000
<30	1,791	6.7 (5.6–7.9)		6.3 (5.2–7.5)	
30–39	1,344	3.4 (2.5–4.5)		4.3 (3.3–5.5)	
40–49	794	2.1 (1.3–3.4)		6.3 (4.7–8.2)	
50+	986	0.7 (0.3–1.5)		2.3 (1.5–3.5)	
*Education (years of schooling)*			p = 0.000		p = 0.000
None	2,335	1.4 (0.97–2.0)		2.7 (2.1–3.4)	
1–5	1,462	2.1 (1.4–2.9)		4.7 (3.7–5.9)	
6+	1,112	11.2 (9.4–13.2)		10.8 (9.0–12.7)	
*Asset quintile*			p = 0.000		p = 0.000
Lowest	1,004	0.1 (0.0–0.6)		2.5 (1.8–3.9)	
2	973	1.4 (0.8–2.4)		2.2 (1.3–3.3)	
3	1,002	1.8 (1.1–2.8)		3.4 (2.4–4.7)	
4	954	3.1 (2.04–4.3)		4.3 (3.1–5.8)	
Highest	981	12.9 (10.9–15.2)		12.5 (10.4–14.7)	

### Use of existing mHealth services

In terms of the actual use of mHealth services, only 11% of the respondents who knew about mHealth services actually called special numbers/call centres to seek treatment and no one actually used the mHealth services provided by upazila health complex (Table 5). Males and those from the richest households reported seeking mHealth services more than the females and those from the poorest households. Interestingly, in terms of education of the respondents, the difference was not evident (Table 5).

**Table 5:** Calling of the mHealth services.

Variable	Number of respondents who knew about the special number	% of respondents called special number (95% CI)	Statistical significance
Ever called	189	11.6 (7.4–17.1)	
*Sex*			p = 0.044
Male	138	14.5 (9.1–21.5)	
Female	51	3.9 (0.5–13.5)	
*Education (years of schooling)*			p = 0.612
None	33	12.1(3.4–28.2)	
1–5	31	6.5 (0.8–21.2)	
6+	125	12.8 (7.5–19.9)	
*Asset quintile*			p = 0.830
Poorest	01	0.0(–)	
2	14	7.1 (0.18–33.8)	
3	18	5.6 (0.14–27.3)	
4	29	10.3 (2.9–27.4)	
Richest	27	13.4 (8.0–20.5)	

### Use of mobile phone and mHealth services for healthcare

**Figure 2:**
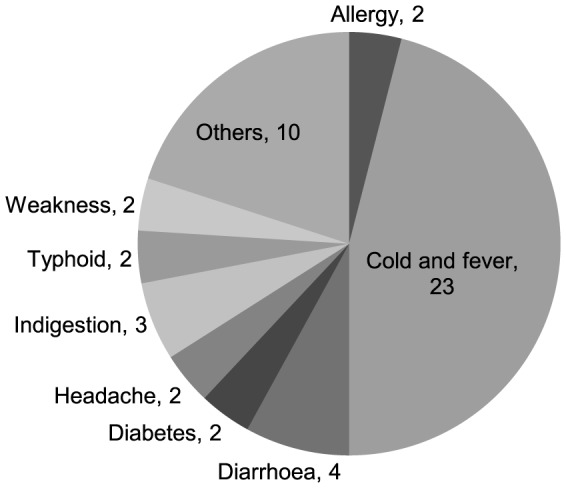
Distribution of patients who contracted doctors using mobile phone by type of disease.

Table 6 presents data on healthcare-seeking behaviour of the household members included in the survey in the case of sickness during two weeks preceding the day of data collection. Total population in the respondents' households was 26,591 at the time of the survey;. 37.7% (10,026/26,591) of the household members had a sickness during two weeks preceding the survey. For 46.83% (4,695/10,026) of those who were sick a contact with a healthcare provider was made. The most contacted healthcare provider was village doctor (57.4%) followed by village homeopath (16.5%), MBBS doctor (15.7%), trained paramedic (7.9%) and *kabiraj/hujur* (2.4%). Based on the sample of one randomly-chosen patient per household from those who contacted a healthcare provider (2,581 patients), it was estimated that 1.9% (50 patients) contacted a provider, using a mobile phone, and 98.1% had a face-to-face consultation. Use of mobile phone was more for female (2.4%) patients than male (1.4%) in all age groups in contacting a healthcare provider for consultation. The highest use of mobile phone (2.3%) was recorded for under-five children, followed by age-group 18+ (2.1%); and the lowest use (0.9%) was observed for the 6–17 years age-group. Cold-and-fever was one of the main sicknesses for which almost half of the patients (23) contacted a doctor, using mobile phone, followed by diarrhoea (4) and indigestion (3) (Figure 2). Use of mobile phone was the highest for contacting a village doctor (58.2%), followed by an MBBS doctor (36.5%), icddr,b doctors and paramedics (4.2%), and homoeopaths (2.1%).

**Table 6:** Health seeking behavior during sickness.

Variable	Denominator	%	95% CI
Sickness during two weeks preceding the survey	26,591	37.70	(37.1–38.3)
Contact with any healthcare provider	10,026	46.83	(45.8–47.8)
Types of healthcare provider contacted:	4,695	100.0	
Village doctor		57.44	(56.0–58.8)
Homoeopath		16.55	(15.5–17.6)
MBBS		15.70	(14.7–16.8)
Trained paramedic		7.86	(7.1–8.7)
Traditional (kabiraj/hujur)		2.45	(2.3–2.9)
Use of mHealth services/mobile phone for healthcare	2,581	1.93	(1.4–2.5)

### Cost of healthcare

The median cost of treatment per patient based on all patients totaling 1886 who sought care was Taka (Tk) 150, with an interquartile range (IQR) of Tk 370 (US$ 1  =  Tk 78). There was no differences in cost by sex but there was a significant difference by age (p<0.01), with higher cost for children under age five years, and older age groups compared to younger groups (Tk 160, Tk 100, and Tk 160 for under-five children, for those aged 6–17 years, and 18 or more years respectively). Although the difference in cost by the nature of consultation though was not statistically significant, the cost was higher in face-to-face consultations compared to consultation through mobile phone (Tk 150 versus Tk 124). Of the 2,581 patients for whom a face-to-face contact with a healthcare provider was made, 71% received advice for drugs. 87% of those who received advice for drugs bought all the drugs, 12% bought some, and 1% did not buy any. Of those who contacted a provider through a mobile phone, 68% had received advice for drugs. Of them, 88% bought all the drugs and 12% did not buy all of the drugs. Sixty-four percent of the patients who consulted using a mobile phone took all the prescribed drugs compared to 75% of those who had face-to-face consultations (Table 7).

**Table 7:** Cost of treatment by background characteristics.

Variable	Median	IQR	Number of respondents who contacted doctor	Statistical significance
*Sex*				*p = 0.40
Male	150	357	869	
Female	150	380	1017	
*Age (years)*				**p = 0.0001
≤5	160	244	397	
6–17	100	210	306	
18+	160	450	1183	
*Way of contact*				*p = 0.09
Mobile	124	248	41	
Face-to-face	150	257	1845	

*Based on Mann-Whitney non-parametric test;

**Kruskal-Wallis rank test [for compliance with prescription]; IQR  =  Interquartile range.

### Intention to use mHealth services

Although the actual use of mHealth services was quite low, 29% of the respondents intended to use mHealth in the future (Table 8). Males, more educated persons, and people from richer households intended to use mHealth in the future more than females, those with no schooling and those from poorer households.

**Table 8:** Intention to use mHealth in the future.

Variable	Number of respondents	% of respondents	95% CI	Statistical significance
Has intention to call in the future	4,081	28.5	(27.1–29.1)	
*Sex*				p = 0.000
Male	1,729	33.6	(31.4–35.8)	
Female	2,352	24. 6	(22.9–26.7)	
*Education (years of schooling)*				p = 0.000
None	1,791	21.6	(18.7–22.5)	
1–5	1,271	27.1	(24.7–29.7)	
6+	1,013	42.4	(39.3–45.5)	
*Asset quintile*				p = 0.000
Lowest	744	17.6	(14.9–20.5)	
2	796	23.4	(20.5–26.5)	
3	832	26.7	(23.7–29.8)	
4	833	30.8	(27.7–34.1)	
Highest	876	41.7	(38.4–45.1)	

### Reasons for using mobile phone

Thirty percent of the respondents wanted to use mobile phone for seeking healthcare because of low cost, followed by time-saving (24%), instant treatment (18%), acquaintance with doctor (10%), when disease is severe (8%), no transportation cost (6%) and other causes (4%) (Table 9).

**Table 9:** Reasons for calling/not calling doctor for future sickness with mobile phones (First answer reported).

Reasons for calling a doctor for future sickness with mobile phones (N = 1,161)	Percentage (95% CI)	Reasons for not calling a doctor for future sickness with mobile phones (N = 2,919)	Percentage (95% CI)
Low cost	28.94 (26.3–31.6)	Don't know which number to call	21.7 (20.2–23.2)
Saves time	23.83 (21.4–26.4)	No idea about health care through mobile	5.39 (4.6–6.3)
Instant treatment	17.94 (15.7–20.2)	Feel shy talk to male doctor	0.42 (0.21–0.71)
No transportation cost	6.42 (5.4–7.9)	Doctor's chamber is nearby	9.45 (8.4–10.6)
Doctor is known	9.76 (8.8–11.6)	Doctor does not receive the call	0.63 (0.36–0.97)
Disease is severe	8.18 (6.7–9.9)	Direct consultation is better	40.67 (38.3–42.5)
Can get treatment at home	1.14 (0.6–1.9)	Don't have mobile phone	2.87 (2.3–3.5)
To know the presence of doctor in chamber	2.20 (1.5–3.3)	Can't explain the medical condition	13.27 (12.1–14.5)
Others	1.58 (0.9–2.4)	Can't take physical examination	3.29 (2.7–4.0)
		Can get treatment, not medicine	0.74 (0.47–1.2)
		Others	1.62 (1.2–2.1)

### Reasons for not using mobile phone

Forty-one percent of respondents reported that they did not want to use mobile phone for healthcare because they thought direct consultation was better, followed by not knowing which number to call (22%), not being able to explain symptoms to doctor over phone (13%), not feeling the need to use mobile phone as doctor's chamber was nearby (9%), not having ideas about availing healthcare through mobile phone (5%), and perceiving that mobile phone consultation cannot replace physical examination (3%) (Table 9).

## Discussion

The findings reflect many important aspects of mHealth services in Bangladesh including their prospects and challenges. To make the best use of the opportunity this technology offers, the potential and challenges should be well- understood to maximize the opportunity in settings similar to Bangladesh. This study focused on finding what may prevent people from using mobile phone services for healthcare, and to identify customers' interests in using mobile phones if the opportunity to use these for healthcare is expanded. To our knowledge this is the first study that systematically explored these aspects of mHealth in rural Bangladesh.

About 80% of the households own a phone, in this impoverished society; it is actually quite impressive and, thus mHealth has the potential to reach a larger portion of the population. The introduction of mobile phones in Bangladesh is not that old perhaps 10 years. The disturbing fact, though expected, is the high level of socioeconomic and gender inequality in the ownership. However, it should be mentioned that although ownership of phones ensures convenience of access to services, not owning one does not necessarily mean that members of these households are in all that disadvantageous position when there is a need to use one. Availability of shops and entrepreneurs selling mobile phone call services is abundant in Bangladesh. The ‘Phone Lady’ was one such initiative which comprises a woman of Grameen Bank borrower who runs a business of selling mobile phones airtime to customers in a flexible manner in terms of timing and physical availability of the services. The ‘Phone Lady’ has the arrangement of going to the client's households at the request of the customer, helping to ensure almost universal access to mobile phones in Bangladesh however remote or poor is the household. The gender differential in ownership may be a real hindrance in seeking healthcare for females and shows the same pattern of gender differentials against females in healthcare-seeking prevalent in this society. Globally, women are 21% less likely to own a mobile phone than men and this gender disadvantage is the highest in South Asia, followed by sub-Saharan Africa [12]. In Kenya, disparities exist in mobile phones ownership with respect to gender, age, education, literacy, urbanization and poverty [13], [14]. The negative relationship between age of the respondents with the ownership should be a lesser problem in terms of restrictive access by the older members of the household because the inherent age hierarchy is in favour of seniors in this society. The higher rate of mobile phone ownership among younger members of a household also means that younger generation is more inclined to modern technology that clearly indicates the future demand of this technology and the potential it has. So is the case for education of respondents for the future generation will be more educated.

The use of mobile phones for health services is dependent on whether people are aware of this aspect of this technology. The low level of knowledge about the use of mobile phone for health services and the perception that only a small proportion of villagers use these can be one of the contributory factors in the low use of mobile phone for healthcare or mHealth services. The findings that educated and upper-class people use these services set this as an aspirational target for the others which is also indicative of a potential large future demand for the use of mobile for health and mHealth services. The higher use of mobile phones for contacting a healthcare provider in case of a female than male patient reflects the influence of the cultural factor that limits female mobility in this rural area; females are only taken out for healthcare when all other options are exhausted.

Although various mHealth services have been in place for quite some time, the level of knowledge about their existence has been low. This is also a factor contributing to the low level of use of the services from the private and public sectors. It is a prerequisite that the availability of these services be widely known for increasing the use of the services.

The high level of contacting village doctors clearly indicates the continuation of dominance of informal health care providers in delivering services to the people in this community. Thus, special mHealth services targeted to these groups of healthcare providers are likely to derive maximum benefit for the people in this community. The favorable environment for the use of mobile phone/mHealth in terms of many advantages has also been reflected in an in-depth investigation recently carried out in the study area [4].

The similar level of compliance with advice received from a face-to-face consultation versus consultation through the use of mobile phone or mHealth indicates the equal value the study population gives to both types of consultation. This is very encouraging and clearly indicates the potential the mobile phone and mHealth service has in ensuring quality services to the people. Therefore, it is evident that the respondents valuing consultation through mobile phones and mHealth has started to earn peoples' trust. It should however be mentioned that the lower cost of consultation though mobile phone compared to face-to-face consultation does not imply the absence of financial barrier because the cost of mobile telemedicine is very high for the rural areas compared to the per-capita income in Bangladesh [15]. There is a need to find ways to reduce the cost of mHealth services in Bangladesh to ensure access for the poor and the disadvantaged population. Toll free mHealth services or free services for people who are in low socioeconomic condition would be one of the options for enhanced uptake [16], [17].

The intention to use mHealth or mobile phone for health services in the future is another indication of the future demand that the use of mobile phone and mHealth has. The findings showed that nearly 70% of the respondents prefer calling a doctor by using a mobile phone for either low cost or time-saving or instant advice. These heavily weigh in favour of future large demand for this technology for healthcare. The major reasons cited by the respondents for not intending to use mHealth services for health care was their belief that direct visits were better than consulting remotely; they comprised only 40% of the respondents. The other reasons has the potential to be appropriately dealt with in favour of mobile phone or mHealth. Our findings are also in line with Akter et al. 2010 who also found that patients' perceived service quality had a strong significant impact on satisfaction and intention to continue usage [18].

The study findings have important implications for designing future mHealth programme in Bangladesh and elsewhere. It should be mentioned that despite encouraging findings from this study in favour of mHealth, the services cannot replace traditional services involving face-to-face consultation; mHealth should rather be viewed as a complimentary mechanisms where trained heath workforce is unavailable or absent. Given the weak health system and lack of awareness about mHealth services, it is now evident that strategy to dissemination of the importance and availability of mHealth is crucial. Success of mHealth will depend on the quality of the services, cost, broadening the service components, which is possible with the recent introduction of 3G in the country. It is important to have regular monitoring of the reach, quality, cost and effectiveness of mHealth services on the use of healthcare and health of the population. Policy-makers can use findings from this and other studies to institute appropriate policy measures to enhance access, utilization and effectiveness of mHealth in a country like Bangladesh.

In conclusion, the environment is quite favorable to the use of mobile phone for healthcare and or mHealth. It is indicative of the potential this technology-based platform has in providing quality healthcare services to the mass people, especially in settings where there is shortage and/or unavailability of trained health workforce. However, it is important to have regulatory framework and monitoring system in place to ensure accountability and quality of services.

## References

[1] World Health Organization (2007) WHO Bangladesh Country Cooperation Strategy 2008–2013. Dhaka, Bangladesh World Health Organization.Country office for Bangladesh.

[2] Bangladesh Health Watch (2008) The state of health in Bangladesh 2007: Health workforce in Bangladesh; Who constitutes the healthcare system?. Dhaka, Bangladesh.

[3] Mahmood SS, Iqbal M, Hanifi SM, Wahed T, Bhuiya A (2010) Are ‘Village doctors’ in Bangladesh a curse or a blessing? BMC International Health Human Rights 3: 10–18. 10.1186/1472-698X-10-18 PMC2910021 20602805

[4] Bhuiya A, Iqbal M (2013) Use of mHealth to improve the quality of services of the village doctors Recent learning from Chakaria, Bangladesh. Dhaka, Bangladesh International Centre for Diarrhoeal Disease Research, Bangladesh.

[5] United Nations foundation & Vodafone foundation (2009) mHealth for Development: The opportunity of mobile technology for healthcare in developing world.

[6] The Earth Institute at Columbia University, Promise. M, (UNDP). TUDP (2010) Millennium villages project The impact of mobile connectivity on the Millennium Development Goals in Africa.

[7] Ahmed T, Bloom G, Iqbal M, Lucas H, Rasheed S, et al. (2014) E-health and M-Health in Bangladesh: Opportunities and Challenges. Brighton, UK: Institute of Development Studies.

[8] Hanifi SMA, Rasheed S, Mamun AA, Urni F, Hoque S, et al. (2011) Chakaria Health and Demographic Surveillance System Focusing on the Poor and Vulnerable. Mohakhali, Dhaka 1212, Bangladesh. ICDDR, B.

[9] National Institute of Population Research and Training (NIPORT), MEASURE Evaluation (2011) Bangladesh Demographic and Health Survey.

[10] Bhuiya A, editor (2009) Health for the rural masses: insights from Chakaria: International Centre for Diarrhoeal Disease Research, Bangladesh.

[11] Wahed T, Rasheed S, Bhuiya A (2012) Doctoring the Village Docotrs. Dhaka, Bangladesh: Iternational Centre for Diarrhoeal Disease Research, Bangladesh.

[12] GSMA Development Fund, Cherie Blair Foundation for Women&Vital Wave Consulting (2010) Women & Mobile: A Global Opportunity. A Study on the Mobile Phone Gender Gap in Low and Middle-Income Countries. London: GSMA, London.

[13] Wesolowski A, Eagle N, Noor AM, Snow RW, Buckee CO (2012) Heterogeneous mobile phone ownership and usage patterns in Kenya. PLoS One 7: e35319. 22558140 10.1371/journal.pone.0035319 PMC3338828

[14] Zurovac D, Otieno G, Kigen S, Mbithi AM, Muturi A, et al (2013) Ownership and use of mobile phones among health workers, caregivers of sick children and adult patients in Kenya: cross-sectional national survey. Global Health 9: 20. 23672301 10.1186/1744-8603-9-20 PMC3695884

[15] Ivatury G, Moore J, Bloch A (2009) A doctor in your pocket: Health hotlines in developing countries,Innovations: Technology, Governance & Globalization. MIT Press Journal (online) 4: 119–153.

[16] Huq NL, Koehlmoos TP, Azmi AJ, Quaiyum MA, Mahmud A, et al (2012) Use of mobile phone: Communication barriers in maternal and neonatal emergencies in rural Bangladesh. International Journal of Sociology and Anthropology 4.

[17] Mobile Alliance for Maternal Action (MAMA) Reaches Moms and Babies in 22 Countries with Critical Health Information.

[18] Akter S, D'Ambara J, Ray P. User Perceived Service Quality of mHealth Services in Developing Countries; 2010 6–9 June; Pretoria, South Africa.

